# How the AHR Became Important in Intestinal Homeostasis—A Diurnal FICZ/AHR/CYP1A1 Feedback Controls Both Immunity and Immunopathology

**DOI:** 10.3390/ijms21165681

**Published:** 2020-08-08

**Authors:** Agneta Rannug

**Affiliations:** Karolinska Institutet, Institute of Environmental Medicine, 171 77 Stockholm, Sweden; Agneta.Rannug@ki.se

**Keywords:** aryl hydrocarbon receptor, butyrate, circadian, cytochrome P4501A1, 6-formylindolo[3,2-*b*]carbazole, gut microbiome, inflammatory bowel disease, innate lymphoid cells, interleukin 22, phytochemicals

## Abstract

Ever since the 1970s, when profound immunosuppression caused by exogenous dioxin-like compounds was first observed, the involvement of the aryl hydrocarbon receptor (AHR) in immunomodulation has been the focus of considerable research interest. Today it is established that activation of this receptor by its high-affinity endogenous ligand, 6-formylindolo[3,2-*b*]carbazole (FICZ), plays important physiological roles in maintaining epithelial barriers. In the gut lumen, the small amounts of FICZ that are produced from L-tryptophan by microbes are normally degraded rapidly by the inducible cytochrome P4501A1 (CYP1A1) enzyme. This review describes how when the metabolic clearance of FICZ is attenuated by inhibition of CYP1A1, this compound passes through the intestinal epithelium to immune cells in the lamina propria. FICZ, the level of which is thus modulated by this autoregulatory loop involving FICZ itself, the AHR and CYP1A1, plays a central role in maintaining gut homeostasis by potently up-regulating the expression of interleukin 22 (IL-22) by group 3 innate lymphoid cells (ILC3s). IL-22 stimulates various epithelial cells to produce antimicrobial peptides and mucus, thereby both strengthening the epithelial barrier against pathogenic microbes and promoting colonization by beneficial bacteria. Dietary phytochemicals stimulate this process by inhibiting CYP1A1 and causing changes in the composition of the intestinal microbiota. The activity of CYP1A1 can be increased by other microbial products, including the short-chain fatty acids, thereby accelerating clearance of FICZ. In particular, butyrate enhances both the level of the AHR and CYP1A1 activity by stimulating histone acetylation, a process involved in the daily cycle of the FICZ/AHR/CYP1A1 feedback loop. It is now of key interest to examine the potential involvement of FICZ, a major physiological activator of the AHR, in inflammatory disorders and autoimmunity.

## 1. Introduction

Although the cytochrome P450 (CYP) family of enzymes was initially thought to catalyze the metabolism of xenobiotics and thereby be involved in chemical carcinogenesis, it has since become clear that many of these enzymes also play physiological roles in the metabolism of a variety of endogenous compounds [1]. One of these endogenous compounds is 6-formylindolo[3,2-*b*]carbazole (FICZ), which binds to the aryl hydrocarbon receptor with the highest affinity yet reported. In stark contrast to the anthropogenic 2,3,7,8-tetrachlorodibenzo-*p*-dioxin (TCDD) and other well-characterized ligands for the AHR, FICZ is also an excellent substrate for CYP1A1, CYP1A2, and CYP1B1, all encoded by genes regulated by the AHR [1]. Accordingly, FICZ participates in an autoregulatory feedback loop, which maintains its own steady-state concentration, like that of many hormones, at a low level (reviewed in [2]).

FICZ was discovered serendipitously in connection with experiments designed to produce dimerized photoproducts of planar biomolecules for testing as ligands for AHR. Adenine was subjected to ultraviolet radiation in the presence of tryptophan (Trp), a photo-sensitizing molecule, and it turned out that similar irradiation of solutions containing Trp alone produced some compound(s) that could compete efficiently with TCDD for binding to the AHR. Two products with molecular weights of 284 and 312 Daltons were identified and immediately assumed to be endogenous signal substances, since they bind to this receptor with higher affinity than any other known compound, including TCDD [3,4].

Subsequently, FICZ was shown to be formed upon exposure of Trp to H_2_O_2_ alone, as well as via several enzyme-catalyzed pathways [5]. To date, FICZ has been detected by liquid chromatography-mass spectrometry in aged batches of Trp [3], cell culture media [6,7], cultured non-hematopoietic cells [8], hematopoietic cells [9], yeast cells [10], and extracts of human skin [10,11], as well as in mouse colorectal tissue [12]. In addition, sulfoconjugates of phenolic metabolites of FICZ are present in human urine [13]. Thus, ubiquitous formation/presence of FICZ, albeit at low levels, in most tissues under normal conditions is highly probable. Still, establishing FICZ as an important endogenous AHR agonist has been controversial since many studies have described the AHR to be highly promiscuous and suggested AHR ligand binding by a myriad of both endogenous and exogenous molecules.

Although the AHR is not essential for survival, this receptor is involved in several physiological processes, including regulation of homeostasis and immunity at epithelial barriers such as the one formed by intestinal epithelial cells (IECs) (reviewed by [14]). Since the most potent immune responses in the body occur in the gut, considerable focus is now being placed on elucidating the molecular mechanism(s) underlying the role of the AHR in cells in the intestinal mucosa—including the IECs and various immune cells, such as B cells, T cell receptor γδ T cells (TCRγδ), T helper 17 cells (Th17), regulatory T cells (Treg), type 1 regulatory T cells (Tr1), innate lymphoid cells (ILC), macrophages (MQ), intraepithelial lymphocytes (IEL), dendritic cells (DC), and neutrophils (reviewed by [15]).

CYP1A1 plays an essential role in the intestinal immune system, controlling related steady-state processes, as well as responses to pathogenic insults. In the present review, I discuss new perspectives on the role of FICZ produced by the microbiota in gut immunity, with particular focus on the key role played by CYP1A1 in the dynamic regulation of FICZ-stimulated production of interleukin 22 (IL-22) and the temporal pattern of AHR signaling in the gut.

## 2. Activators of the AHR Promote Intestinal Immune Responses

The enormous numbers and huge variety of bacteria in the intestine, as well as the pronounced diurnal oscillations in both the composition and function of the gut microbiome, require an efficient barrier for protection of the host. Normally, abundant secretion of mucus and a vigorous, but closely regulated immune system maintain this barrier, but perturbations of gut microbiota (termed dysbiosis) are associated with several pathological states, including inflammatory bowel disease (IBD), metabolic syndrome, and colorectal cancer (CRC) (reviewed in [16]).

Recently, several important investigations have established that the AHR is required both for regulation of the homeostasis of the intestinal epithelial and associated immune cells, as well as for mounting appropriate responses to epithelial damage and invading pathogens [17,18,19,20,21,22]. In addition, this receptor appears to be involved in peristaltic and secretory reflexes [23,24].

In mice raised in a conventional manner, CYP1A1 is expressed by epithelial cells in the duodenum, jejunum and ileum [25], with the most pronounced up-regulation by TCDD occurring in the proximal parts of the small intestine (SI) [26]. Mice that are germ-free (GF) or have been treated with an antibiotic and are thus exposed to lower levels of factors produced by the microbiota, express the AHR, AHRR, and CYP1A1 genes at lower levels in their SI [25,27,28,29]. The lack of functional AHR signaling in such animals may explain why they are more susceptible to colitis induced experimentally, e.g., by trinitrobenzene sulfonic acid (TNBS) or dextran sulfate sodium (DSS) [30,31]. Such findings highlight the involvement of commensal microbes in the intestinal immune system through their production of factors that activate the AHR.

### 2.1. Dietary Activators of the AHR

Even before Alan Poland described the AHR in 1976 [32], Lee Wattenberg and colleagues had reported an elevated level of CYP1A1 activity (at that time measured as benzo[a]pyrene (BaP) metabolism or aryl hydrocarbon hydroxylase (AHH) activity) in the liver and gastrointestinal tract (GI) of rodents fed standard chow [33]. They discovered subsequently that feeding rats and mice cereal-based chow and synthetic semi-purified diets fortified with phytochemicals isolated from the *Brassicaceae* family of plants, alfalfa or spinach resulted in a high basal level of highly inducible CYP1A1 activity in their gut, from the gastrointestinal epithelium of the SI to the colon (reviewed by [34]). This basal activity was most pronounced in the proximal region of the SI.

These findings, in combination with the early interest in phytochemicals as potential anti-carcinogens, motivated a number of investigations designed to test whether cruciferous vegetables induce AHH activity. For example, indole-3-carbinol (I3C), a hydrolysis product of glucobrassicin produced by myrosinase, was found to increase CYP1A1 activity in the liver and intestine of rodents [35,36]. I3C itself binds to the AHR with low affinity, but under acidic conditions can give rise to indolo[3,2-*b*]carbazole (ICZ), which has high affinity [37].

Investigations designed to unravel the mechanisms by which dietary phytochemicals induce CYP1A1 in the gut and also influence gut immunity and protect against inflammatory disorders within the GI often involve comparisons between conventional chow based on grain and purified diets (AIN-76, AIN-93, or similar) supplemented with I3C. It is now known that a wide range of phytochemicals—including flavonoids, alkaloids, stilbenes and curcuminoids—bind to this receptor with no or low affinity but still activate CYP1A1, and we have proposed that compounds that inhibit the expression and/or, activity of CYP1A1 may attenuate clearance of endogenous FICZ, leading to indirect activation of the AHR [38]. This may explain why this receptor in the intestine, systemically, is activated by many dietary phytochemicals that are potent inhibitors of CYP1A1 activity [39,40].

### 2.2. Microbial Activators of the AHR

The symbiosis between resident microbiota and the host is beneficial to both in many ways. Under the anaerobic conditions in the gut, the microbiota modify many small molecules present in the diet and these then enter the host circulation and influence immunity and other distal functions in a manner that contributes to the well-being of the host. For instance, gut bacteria give rise to essential vitamins (e.g., A, group B and K), as well as serotonin (5-HT), short-chain fatty acids (SCFAs), and some secondary bile acids [41,42].

These microbiota also metabolize the essential amino acid Trp, present at high levels in protein-rich food, into a multitude of different indoles. Examples include indole itself, indole-3-acetaldehyde (IAAl), indole-3-acetic acid (IAA), indole-3-aldehyde (IAl), indole-3-lactic acid (IAL), indole-3-acrylic acid (IA), indole-3-propionic acid (IPA), indole-3-pyruvate (I3P), skatole, and tryptamine (Tra). Many of these activate AHR signaling (reviewed by [42,43,44,45]).

It is now evident that indole metabolites of Trp formed in the mammalian intestine play important roles in gut health. In particular, Trp metabolism by *Lactobacillus* species elicits an anti-microbial immune response including production of IL-22, which is vital for maintenance of mucosal barrier integrity within the SI. Numerous studies with GF rodents or animals treated with antibiotics have demonstrated that reduced microbial catabolism of dietary Trp results in higher concentrations of this amino acid in the serum and or feces and reduced levels of AHR-activating compounds [20,28,46,47,48].

Impaired Trp transport systems also have negative consequences for the health of the gut. For instance, mice lacking the angiotensin-converting enzyme 2 (Ace2) take up Trp from the diet poorly and also display an altered intestinal microbiome and enhanced susceptibility to the development of colitis [49]. Similarly, the intestinal microbiome in mice that lack the caspase recruitment domain family member 9 (CARD9) is altered and these animals fail to metabolize Trp into activators of the AHR, exhibit defective expression of IL-22 and antimicrobial peptides (AMPs) such as RegIIIβ and RegIIIγ in the colon, and are more susceptible to colitis [19]. Furthermore, defects in the LAT1-CD98 complex, which transports aromatic amino acids, in immune cells limit the intracellular level of FICZ due to lower access to Trp [9]. In contrast, the elevated levels of Trp resulting from the reduced metabolism of this amino acid in knock-out mice that do not express indoleamine 2,3-dioxygenase 1 (IDO1) enhances formation of microbial indoles that activate the AHR [20,48].

A recent update identified I3P as one of the microbial catabolites of Trp that activate the AHR most potently [50]. In a competitive binding assay, I3P displaced TCDD from the mouse AHR with an IC_50_ of 55 µM, i.e., more efficiently than IAl (IC_50_ > 1 mM), but much less efficiently than FICZ (IC_50_ 2nM), which was included in this experiment as a positive control, but not considered to be a potential microbial catabolite. However, in another recent study, the levels of FICZ in colorectal mouse tissue were quantified and the results support the notion that this ligand is the most potent AHR-activating Trp catabolite in the intestine [12]. These highly preliminary data require confirmation in further studies.

**Figure 1:**
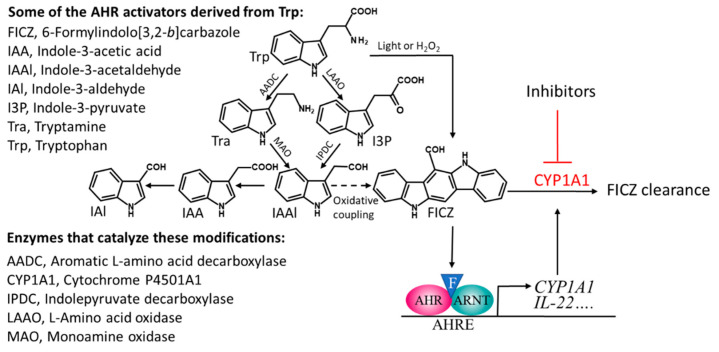
A scheme describing the microbiota-mediated catabolism of tryptophan that leads to several compounds that can activate the aryl hydrocarbon receptor (AHR), including the high affinity ligand FICZ (illustrated by an F in a blue triangle). Stimulation with FICZ causes AHR to partner with the nuclear translocator of AHR (ARNT), bind to AHR response elements (AHREs), and stimulate expression of CYP1A1, which takes part in the metabolic clearance of FICZ. The oxidative coupling that generates FICZ from IAAl has been described in [5].

The species of commensal bacteria that have so far been shown to produce indoles that activate the AHR include certain strains of *Lactobacillus*, *Allobaculum*, *Peptostreptococcus*, and *Propionibacterium* [20,48,51,52]. However, many other such species are likely to be identified, since the common bacterial inhabitants of the gut, belonging to several different phyla, convert Trp into I3P, Tra, or IAAl [42,44,53], all of which are precursors of FICZ and other AHR-activating indoles as well (Figure 1).

Another group of AHR activators produced by the gut microbiota encompass the SCFAs acetate, propionate and butyrate (BUT), of which the role of BUT in maintaining intestinal immune homeostasis is most well documented. BUT is derived from microbial fermentation of indigestible polysaccharides, in particular dietary fibers and resistant starch, which escape digestion by enzymes in the upper gut and are consequently present at relatively high levels (mM) in the lumen of the lower gut (reviewed in [54]). In addition to providing an important source of energy for colonocytes, BUT inhibits inflammation of the intestine and promotes the development and function of Tregs in a beneficial manner. Many of the anti-inflammatory properties of BUT reflect its inhibition of histone deacetylases (HDACs) and simultaneous activation of certain G-protein-coupled receptors on colonic epithelial cells, in particular GPR109A, which is expressed at very high levels by innate immune cells and on the epithelium [55,56,57,58].

Reports published as early as 1996 and 1999 demonstrated that both BUT and trichostatin A, another inhibitor of HDAC, successfully restore expression of the AHR in hepatoma cells deficient in induction of CYP1A1 mRNA, as well as de-repress CYP1A1 expression in fibroblasts non-responsive to ligands of the AHR [59,60]. Several subsequent investigations established that BUT alters expression of the CYP1A1, AHR, and AHRR genes and induces CYP1A1 activity both in different types of cells in vitro [61,62,63,64] and in experimental animals [28,65] by inhibiting HDAC activity. However, although Marinelli and colleagues could reproduce the HDAC-dependent activation of CYP1A1 by trichostatin A, they did not obtain the same effect with BUT, but instead proposed that this compound induced the transcription of AHR-dependent genes as an AHR ligand [66]. Moreover, two other reports documented an increase in the expression of CYP1A1 in cells exposed to BUT alone [62,63]. These latter findings could reflect the presence of FICZ or some other activator of the AHR in the cell culture medium, since, indeed, FICZ has been detected in cell culture media exposed to light [6].

## 3. The Mechanism(s) by Which FICZ, IL-22 and Butyrate Promote Gut Homeostasis

Although dietary and microbial indoles that activate the AHR and support colonization by commensal bacteria are necessary for intestinal health, a balance between tolerance to such beneficial bacteria and immunological responses to potential pathogenic species must be maintained [67,68]. In this context, accumulating evidence indicates that the intestinal epithelium requires a constant supply of moderate levels of IL-22, IL-17, and a granulocyte macrophage-colony stimulating factor (GM-CSF) to protect against undesirable microbial invasion [69].

In adult mammals, the group 3 ILCs (ILC3s) are involved in protecting against microbial pathogens, as well as in regulating the integrity of the intestinal barrier and the relative abundance of the populations of various commensal bacteria. At the same time, through their expression of the transcription factor RORγt and cytotoxicity receptor NKp46 (NKp46^+^RORγt^+^ ILC3s), these cells are considered to be the most important source of IL-22 in the SI lamina propria [69,70,71,72], where they respond to cues provided by the diet and the commensal microbiota, as well as to their own intrinsic circadian rhythm to produce IL-22 [73,74]. In this connection, a key observation was that the AHR is required for postnatal expansion of such intestinal ILCs [19,75,76]. Indeed, Qiu and colleagues have proposed that in mouse pups after weaning, the AHR responds to bacteria in the gut in a manner that leads to the development of RORγt^+^ ILCs [19].

Furthermore, it is well documented that under steady-state conditions, phytochemicals present in conventional rodent chow promote and sustain IL-22-producing RORγt^+^ ILCs [19,75,76,77]. Accordingly, the microbial flora, the AHR, and ligands of this receptor that stimulate IL-22 production by intestinal ILC3s are important innate effectors of intestinal health.

**Figure 2:**
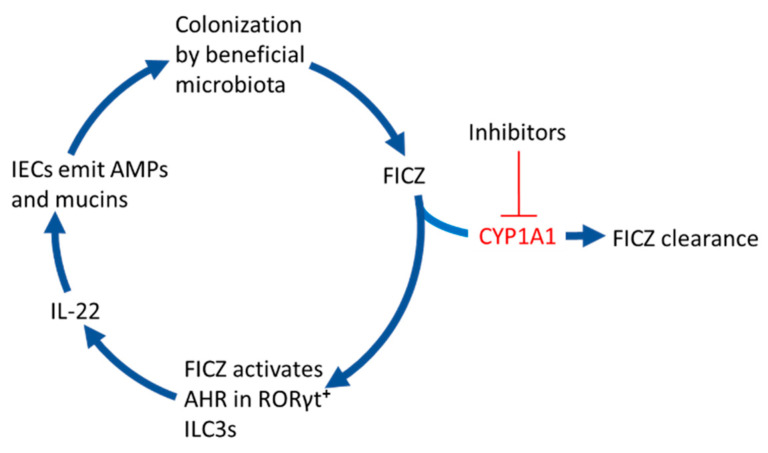
6-formylindolo[3,2-*b*]carbazole (FICZ) stimulates gut colonization by beneficial microbiota. When the CYP1A1-mediated clearance of microbially produced FICZ in the intestinal epithelium is inhibited by for example dietary phytochemicals, drugs, or inflammatory mediators, this compound will bind to the aryl hydrocarbon receptor (AHR) in RORγt positive group 3 innate lymphoid cells (ILC3s). This stimulates them to secrete the interleukin IL-22 that signals to intestinal epithelial cells (IECs) to emit antimicrobial peptides (AMPs) and mucins, which promote colonization by commensal bacteria.

Figure 2 and the sections below describe how the FICZ that is not broken down in the IECs can support colonization of the gut by beneficial strains of bacteria.

### 3.1. Repressors of CYP1A1 Prevent the Clearance of FICZ

The importance of a functional AHR-dependent pathway in the intestinal epithelium for induction of CYP1A1 expression by a factor originating in the gut was first demonstrated by the findings by Ito and colleagues in 2007 [78]. In mice (fed standard chow), where the ARNT gene was disrupted, predominantly in IECs, the levels of CYP1A1 mRNA and corresponding enzymatic activities were markedly elevated in almost all other tissues. In another study performed with mice deficient in CYP1A1/1A2/1B1, endogenous AHR ligands that escape breakdown in the epithelial cell lining activate this receptor, as demonstrated with a *Cyp1a1* fate-reporter [21].

In addition to such genetic silencing, several types of agents that could prevent the CYP1A1-mediated breakdown of FICZ have been described. CYP1A1 activity can be inhibited by external factors consumed orally or formed by microbiota, including dietary phytochemicals such as *α*-naphthoflavone, *β*-naphthoflavone, galangin, chrysin, kaempferol, apigenin, baicalein, and quercetin [40], as well as I3C and its acid-condensation products 3,3-diindolylmethane (DIM) and ICZ [21]. In fact, FICZ can slow down its own metabolic degradation by inhibiting CYP1A1 activity [79]. Other inhibitors of CYP1A1 activity include a large number of anti-inflammatory, anti-depressant, anti-parasitic, anti-psoriatic, beta-blocking, and cytostatic drugs, and inhibitors of proton pumps [38,39] and several ubiquitous carcinogenic polycyclic aromatic hydrocarbons (PAHs), exemplified by BaP and 3-methylcholanthrene (with IC_50_ values in the nM range) [80]. In the studies referred to above, the ability to inhibit 7-ethoxyresorufin *O*-deethylation activity was used as a measure of CYP1A1 inhibition, and the inhibitors were mostly substrates that competed with 7-ethoxyresorufin for binding to the enzyme. Thus, the inhibitors of CYP1A1 that has been shown to attenuate metabolic clearance of FICZ include a wide range of phytochemicals [81], environmental pollutants [82], metals, and oxidants [38,83,84].

Moreover, signals released in connection with microbial-associated molecular patterns (MAMPs) or pathogen-associated microbial patterns (PAMPs) can interact with pattern recognition receptors (PRRs) on the surface of IECs, DCs and MQs to trigger downstream signaling cascades. This promotes the production of mediators of inflammation or infection, such as IL-1β, IL-6, TNFα, and IFNs, which also leads to inhibition of CYP1A1 expression and/or activity (reviewed in [85]).

Theoretically, several molecular mechanisms can be involved in the downregulation of the expression and/or activity of CYP1A1, e.g., competitive or mixed inhibition of the enzyme, alterations caused by reactive oxygen species, or chromatin remodeling in the promoter region of this gene. Regardless of the mechanism involved, lower CYP1A1 activity permits higher concentrations of FICZ to reach innate lymphoid cells that express IL-22.

### 3.2. FICZ Induces Expression of IL-22 by ILC3s

IL-22 binds to the heterodimeric receptor IL-22Rα1/IL-10Rβ on IECs and induces a downstream signaling cascade that leads ultimately to phosphorylation of the transcription factor STAT3. This signaling supports the epithelial barrier against bacterial infections by stimulating secretion of AMPs, e.g., RegIIIβ, RegIIIγ, and members of the S100 family of proteins by enterocytes and Paneth cells in the SI and enterocytes in the colon of mice [86,87]. Paneth cells sense commensal bacteria in the gut and aid in broad regulation of both commensal and pathogenic bacteria that maintain intestinal homeostasis [88,89]. Furthermore, IL-22 controls tissue regeneration and repair through direct action on epithelial stem cells [90], as well as inducing epithelial goblet cells to secrete more components of mucus to form a thick gel-like layer impenetrable to many commensal bacteria, thereby limiting their potential to cause inflammation [91].

As mentioned above, RORγt^+^ ILCs appear to be the principal source of IL-22 under steady-state conditions and their constitutive expression of this cytokine is apparently unaffected by the proinflammatory IL-23 cytokine, which is known to activate IL-22 production as part of the response to pathogens [92]. The strict dependence of the ILC3s and their secretion of IL-22 can be explained by the presence of AHR-responsive elements in the promoter region and intron 1 of the IL-22 gene [19]. Moreover, recruitment of the transcription factor RORγt to the IL-22 promoter is facilitated by the AHR [19,93]. Intriguingly, circadian regulation of the numbers of these cells and their expression of circadian clock genes, AHR and IL-22 was recently documented [73].

The fact that exposure of T cells to FICZ, under conditions that induce Th17-cells, potently up-regulates their level of IL-22 mRNA, was reported in 2008 [94,95]. Subsequently, expression of this same mRNA by differentiated mouse Th17 cells was found to be elevated in the presence of as little as 10 pM FICZ, and this induction was enhanced considerably by co-exposure to fluoranthene, pyrene, and phenanthrene, environmental PAHs that inhibit CYP1A1 [21,82].

It is now clear that FICZ stimulates the expression of IL-22 by a variety of different immune cells, including ILC3s, both in vitro [21,82,96,97,98], including human intestinal lamina propria mononuclear cells from IBD patients [18], and in experimental animals [18,20,29,98,99]. In this context, Monteleone and colleagues (2011) found that a remarkably low amount of FICZ ameliorates colitis induced experimentally in mice [18]. A single intraperitoneal administration of 1 µg per mouse (50 µg/kg) lowered the mortality, enhanced the level of IL-22 in colonic samples, and ameliorated colitis induced by TNBS or DSS. As proof-of-concept, they also demonstrated that a neutralizing antibody against IL-22 largely prevented these anti-inflammatory effects of FICZ.

Similar results were obtained in another study [20], and in their comparable investigation, Zelante and coworkers demonstrated that administration of IAl (18 mg/kg daily for 12 days) to mice ameliorated DSS-induced colitis [48]. In fact, there are nine more reports that FICZ protects against bacterial infections and colitis caused by T-cell transfer, DSS or TNBS [29,99,100,101,102,103,104,105,106], with only one study observing no protection against TNBS-induced colitis [12]. When Qiu and colleagues injected mice intraperitoneally with 0.5 µg FICZ for 6 days, IL-22-producing ILCs accumulated both in the large intestine (LI) and, especially, in the SI [19].

### 3.3. IL-22 Promotes Colonization by Commensal Bacteria

Normally, more than 1 × 10^13^ bacteria, predominantly of the *Firmicutes* and *Bacteroidetes* phyla, symbiotically colonize the mammalian intestine. Microbial density is lowest in the SI and the microbiota demonstrate diurnal rhythmicity with respect to both their localization and production of metabolites that depend on light exposure, the time of food consumption and the type of food [16,107,108,109].

IL-22 has been shown to be needed to promote the colonization of the GI by beneficial bacteria and gut homeostasis both in studies with mice lacking this cytokine and mice treated with antibodies against it [110]. Zenewich and co-workers found that healthy IL-22 KO mice have an altered colonic microbiome containing lower relative abundances of certain families of bacteria, including *Lactobacillaceae*, *Bacteroidaceae*, *Clostridiaceae*, and *Peptococcaceae*, and more of others. In addition, when colitis was induced experimentally into these animals, they developed more severe disease. Moreover, when their altered gut microbiota were transferred to wild-type (WT) mice in the same cage, these wild-type animals also exhibited enhanced susceptibility to experimental colitis [110].

Notably, bacteria that metabolize Trp are more abundant in the SI, where constitutive expression of IL-22 helps to shape and constrain the commensal community [20,111]. It has been proposed that colonization by bacteria that metabolize Trp and/or promote health in other ways is regulated by the availability of mucins, as well as by antimicrobial proteins that may increase the proportion of *Lactobacillus* [20,48,110].

In comparison to the diverse microbiota of mice reared on conventional grain-based chow, the immune phenotype of the microbiome of mice fed purified, phytochemical-free diets (often termed AHR ligand-free diets) is changed in a manner similar to that seen in AHR-deficient animals [28,112,113]. Like the AHR-deficient mice, animals fed purified, phytochemical-free diets exhibit enhanced susceptibility to severe colitis [113,114,115]. Furthermore, there are numerous reports that phytochemicals—including berberine [116], curcumin [117], galangin [118], resveratrol [119], and rutin [120]—can prevent microbial dysbiosis and stimulate colonization of the gut by beneficial anaerobic bacteria (reviewed by [121,122]). 13C, which is commonly used to activate the AHR and stimulates IL-22 expression, can also promote colonization by beneficial bacteria, both when administered in the diet [113] and injected intraperitoneally [123]. Accordingly, Schanz found that purified diets exert a profound negative impact on the composition of the microbiome of the murine SI and colon, lowering counts of Gram-positive bacteria belonging to the *Firmicutes* phylum, such as *Clostridium butyricum, Faecalibacterium prausnitzii, Roseburia,* and *Lactobacillus* species [113]. Importantly, this deleterious change could be reversed by addition of I3C to the diet. Interestingly, the beneficial effects of 13C even after intraperitoneal administration [123] exclude the possibility that DIM and ICZ, acid condensation products of this compound that are formed in the stomach [37], were the protective compounds.

Furthermore, BUT concentrations were higher in mice fed conventional chow diets than in mice fed purified diets [124], and butyrate-producing *Roseburia* spp. were more common in mice fed purified diets and administered I3C intraperitoneally [123], which illustrates the important role of phytochemicals in supporting the growth of BUT-producing bacteria. The lower portion of the SI (ileum) and colon, where microbial density is highest, contain large numbers of BUT-producing bacteria belonging to the *Firmicutes* phyla (e.g., the *Bacteroides fragilis* and *Clostridium clusters IV* and *XIVa* strains) [55,125]. Commensal bacteria that produce BUT and utilize mucins as an energy source are capable of penetrating the inner mucus layer and stimulating the underlying IECs to produce mucin peptides, as well as of supporting the production of different SCFAs. For example, acetate that contributes to the production of BUT must sometimes be supplied through co-colonization by primary fiber degraders that initiate the utilization of complex fibers [54].

The observations described above indicate that conventional chow and diets enriched in phytochemicals influence the composition of the intestinal microbiota in a manner that favors the production of both FICZ and BUT. As expected, this production of beneficial microbial metabolites is reduced in germ-free animals [20,28,46,47,48].

### 3.4. BUT Fine-Tunes IL-22 Signaling

BUT helps maintain immunological homeostasis in the gut by inducing the differentiation of IL-10-producing Treg cells [55] and Tr1 cells [126], in addition to its indispensable counteraction of the pro-inflammatory responses associated with the signaling pathways involving activated nuclear factor-kB (NF-kB) and IL-6/STAT3/IL-17 [127,128,129]. IL-10-producing Tregs play a particularly important role in limiting inflammatory responses and there are relatively high numbers of these cells in the lamina propria (LP) of the ileum and the colon, where the density of BUT-producing bacteria is also higher than in the SI [108]. BUT induces the differentiation and expansion of Tregs by inhibiting HDACs, which results in acetylation of the histones associated with the promoter region of the forkhead box P3 (FoxP3) gene [55]. Differentiation of FoxP3-negative Tr1 cells is enhanced by this same inhibition in combination with signaling through GPR109A [126].

**Figure 3:**
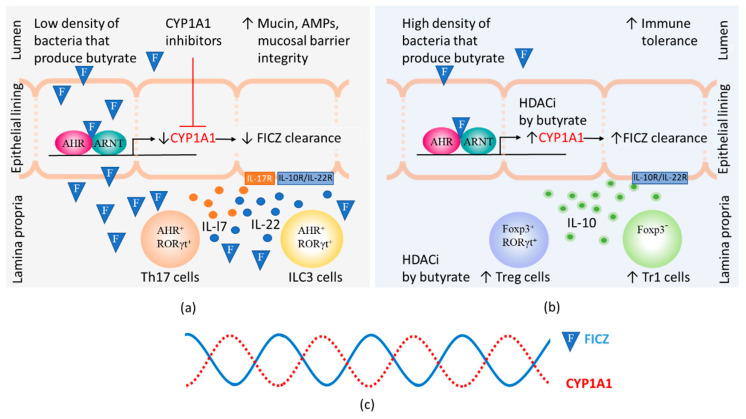
The steady-state levels of 6-formylindolo[3,2-*b*]carbazole (FICZ) influences gut immunity. The amount of FICZ (illustrated by an F in a blue triangle) that reaches the lamina propria depends on the CYP1A1 activity in the epithelial lining, which is modified by the presence of CYP1A1 inhibitors. Butyrate can by virtue of its histone deacetylase inhibitory function (HDACi) contribute to increased numbers of Foxp3 positive and negative regulatory T cells (Treg and Tr1 cells) as well as increased CYP1A1 expression in intestinal epithelial cells. (**a**) At low levels of butyrate, heightened immunity is promoted by CYP1A1 inhibitors that cause FICZ to reach lamina propria and stimulate IL-22 and IL-17 expression in Th17 and ILC3 cells. Binding of IL-17 and IL-22 to their receptors on epithelial cells stimulate increased production of mucins and antimicrobial peptides (AMPs); (**b**) At high levels of butyrate, high CYP1A1 activity promotes the clearance of FICZ. Immunity is suppressed and Treg and Tr1 cells produce IL-10 that helps maintain tolerance to commensal bacteria by binding to receptors on epithelial cells; (**c**) Daily 24 h cycles in the CYP1A1 activity and the levels of FICZ.

When the density of bacteria that produce BUT is low, inhibitors of CYP1A1 activity can elevate the steady-state level of FICZ to which the LP is exposed, thereby stimulating the production of IL-22 by RORγt^+^ ILC3s (Figure 3a). When BUT-producing bacteria are abundant, inhibition of HDAC by BUT enhances the expression and thereby activity of CYP1A1 (for references see Section 2.2). Crucially, this results in more extensive clearance of FICZ, preventing this substance from continuing to stimulate IL-22 production by ILC3s (Figure 3b).

## 4. Diurnal Rhythmicity in CYP1A1 Activity

The findings of Schiering and colleagues linked an absence of CYP1A1 activity in murine IECs to elevated numbers of ILC3s and Th17s in the colon, more IL-22 protein in cultures of colon explants, and an increased response to pathogens [21]. In contrast, the colon of mice whose IECs expressed CYP1A1 constitutively contained substantially fewer ILC3s and Th17s and less IL-22 protein, and these animals were more susceptible to enteric infection. These observations demonstrate that, at least in mice, CYP1A1 activity in IECs control gut levels of IL-22, and that to avoid inflammatory disorders, these levels must be regulated.

Interestingly, there are pronounced diurnal fluctuations in the composition of the intestinal microbiome [130], with as much as a 10-fold difference in the number of bacteria that adhere to the intestinal epithelium at night than day. Moreover, the amounts of SCFAs and other microbial products fluctuate in response to the nature and timing of the diet [130,131]. Consequently, microbial production of ligands derived from Trp and of BUT in the intestine varies during the 24-h day. Moreover, the level of mRNA encoding and activity of CYP1A1 in the liver and lungs of rodents also oscillate during the day [132,133], as do hepatic levels of AHR and ARNT mRNA and protein [133,134], as well as AHR mRNA in enteric ILC3s [73]. However, since few researchers in this field are aware of these fluctuations, few experimental studies include sampling at different times throughout the day.

In summary, a diurnal oscillation in the FICZ/AHR/CYP1A1 autoregulatory loop aids colonization of the gut by a symbiotic microbiome, helping to maintain tolerance to beneficial bacteria (Figure 3c).

## 5. When the Microbial Homeostasis in the Gut Is Disrupted

The fact that IL-22 production by ILC3s has both beneficial and deleterious effects has led several reviews to refer to IL-22 as a two-faced cytokine or a double-edged sword [67,69,74,111]. Under healthy conditions, IL-22 is constitutively expressed by ILC3s in the SI independently of IL-23 [92,111] and barely detectable in the colonic mucosa [91,104,135].

In order to keep pathogenic microbes such as *Citrobacter rodentium* under control in the colon, expression of AMPs by epithelial cells is induced via a process involving IL-23 signaling and early production of IL-22, with subsequent expression of IL-17 that acts synergistically with IL-22 [67,69,70,136]. Qiu and colleagues (2012) found that AHR-deficient ILCs lack the IL-23 receptor (IL-23R) and that AHR KO mice express IL-22 at reduced levels and, unlike wild-type mice, succumb to infection with *C. rodentium*. Administration of a plasmid encoding IL-22 protects such AHR KO animals from early mortality [19]. In line with this, ILC3s in the colon of patients suffering from IBD are dysregulated and express abnormally high levels of both IL-22 and IL-17 [69]. An important observation that has been repeatedly seen is that IBD patients display dramatically augmented microbial dysbiosis, in particular with a reduced abundance of bacteria that produce BUT [137,138,139]. A significant decrease of butyrate production has also been documented to occur in patients suffering from other autoimmune diseases such as type 2 diabetes [140], Behçet syndrome [141], and rheumatoid arthritis [65].

Together, the data documented in Table 1 suggest that, during infections, control of endogenous AHR signaling is required, because otherwise excessive and sustained production of IL-22 may exert deleterious effects on the colon that could lead to chronic inflammatory disorders and potential autoimmunity.

## 6. Conclusions

The observations described above indicate that dietary phytochemicals activate AHR-dependent immune processes, not as ligands to the AHR, but by influencing the composition of the intestinal microbiota in a manner that favors the production of both FICZ and BUT.

Unfortunately, the thousands of experimental studies on colitis that have been reported—involving the suffering and distress of large numbers of laboratory animals—had not yet led to effective treatment of patients with IBD. Novel strategies based on our knowledge concerning the involvement of FICZ in intestinal immunity may lead to more effective control of this disease, as well as of other autoimmune diseases, since FICZ can activate tolerogenic T cells (reviewed in [2]), in addition to the stimulation of systemic IL-22 signaling described in this review. However, as mentioned above, the biological functions of IL-22 are complex and therefore more mechanistic information is needed.

The most striking insight that comes from earlier work in our own laboratory in combination with the literature reviewed here is that rhythmic variations in the levels of FICZ and IL-22 are required for the maintenance of gut homeostasis.

- When CYP1A1 activity is too low (resulting in high levels of FICZ), defenses against commensal and pathogenic microbes are boosted.
- On the other hand, when CYP1A1 activity is too high (low FICZ levels), the host becomes susceptible to infections.
- Diurnal fluctuations in CYP1A1 activity fine-tune the activity of IL-22.

Therefore, a more detailed understanding of the diurnal regulation of crosstalk between FICZ and commensal bacteria that produce BUT, both when the intestinal barrier is functioning normally and during periods of infection, could pave the way for novel therapies for IBD, other autoimmune diseases, and possibly, for CRC as well.

## Figures and Tables

**Table 1:** The potential influence of various conditions that regulate the level of CYP1A1 activity on the levels of FICZ and immunity in the intestine.

Condition	Level of FICZ	Level of IL-22	Intestinal Immunity	Impact on Health
Conventional diets	Depends on timing of food intake	Fluctuating	Balanced	Normal
Purified diets	Low	Low	Low	Immunosuppression
GF or antibiotic treatment	Low	Low	Low	Immunosuppression
Constitutively high CYP1A1 activity	Low	Low	Low	Immunosuppression
Constitutively low CYP1A1 activity	High	High	High	Inflammatory disorders and autoimmunity

## Abbreviations

AHR	aryl hydrocarbon receptor
AHRE	AHR response element
AHRR	AHR repressor
ARNT	nuclear translocator of AHR
AMP	antimicrobial peptides
BUT	butyrate
CRC	colorectal cancer
DC	dendritic cell
DIM	3,3-Diindolylmethane
DSS	dextran sulfate sodium
FICZ	6-Formylindolo[3,2-*b*]carbazole
GF	germ-free
GPR109a	G-protein coupled receptor *109a*
HDAC	Histone deacetylase
IA	indole-3-acrylic acid
IAA	indole-3-acetic acid
IAAl	indole-3-acetaldehyde
IAl	indole-3-aldehyde
IAL	indole-3-lactic acid
I3C	indole-3-carbinol
I3P	indole-3-pyruvate
IBD	inflammatory bowel disease
IC_50_	half maximal inhibitory concentration
ICZ	indolo[3,2-*b*]carbazole
IEC	intestinal epithelial cells
IEL	intraepithelial lymphocytes
IL	interleukin
ILC3	group 3 innate lymphoid cells
IPA	indole-3-propionic acid
KO	knock out
LI	large intestine
LP	lamina propria
MQ	macrophages
RORγt	RAR-related orphan receptor γt
SCFA	short chain fatty acid
SI	small intestine
STAT	signal transducers and activator of transcription
TCDD	2,3,7,8-tetrachlorodibenzo-*p*-dioxin
TCR	T cell receptor
TGF-β	tumor growth factor beta
TNBS	trinitrobenzene sulfonic acid
Tra	tryptamine
Tr1	type 1 regulatory T cells
Treg	regulatory T cells
Trp	tryptophan
UC	Ulcerative colitis
WT	wild type
